# Serratus Anterior Plane Block as a Primary Anesthetic Technique for Video-Assisted Thoracic Surgery in a Child

**DOI:** 10.7759/cureus.15283

**Published:** 2021-05-27

**Authors:** Caroll N Vazquez-Colon, Ayodele Oke, Alberto Rivera-Cintron, Angela C Lee

**Affiliations:** 1 Anesthesiology, Pain and Perioperative Medicine, Children's National Hospital/George Washington University, Washington DC, USA; 2 Anesthesiology and Critical Care Medicine, Children's Hospital of Philadelphia, Philadelphia, USA

**Keywords:** cystic fibrosis, serratus anterior plane block, regional anesthesia, pediatric anesthesiology, video assisted thoracoscopic surgery

## Abstract

Cystic fibrosis (CF) commonly affects those of European descent; however, it can also be found in those of Asian, African, and Caribbean descent. Patients with CF may have significant lung disease, and their perioperative management can be challenging for the anesthesiologist. In this case report, we describe the use of serratus anterior plane block (SAPB) and IV sedation as an alternative to general anesthesia with an endotracheal tube in a patient with CF pulmonary exacerbation presenting to the operating room for a video-assisted thoracic surgery (VATS).

## Introduction

Cystic fibrosis (CF) is an autosomal recessive genetic disorder that results from a mutation in the CF transmembrane regulator (CFTR) gene on chromosome 7 [1-2]. CF has been traditionally defined as the most common life-threatening inherited disorder of children in Caucasian populations, with an incidence of 1/2500 live births [3]. The prognosis of CF patients has greatly improved in recent decades. One of the most striking pieces of evidence of this change has been the substantial growth in the proportion of adult patients, which currently exceeds 50% in most countries and even 60% in Canada [3]. Patients typically present with recurrent pulmonary infections, elevated sweat chloride levels, and pancreatic insufficiency. Respiratory complications include productive cough, pneumonia, hypoxemia, viscous secretions, hyperinflation of the lung, obstructive lung disease, and pneumothorax. Despite advances in CF management, pulmonary exacerbations continue to be a hallmark of this disease. The estimated median age of survival of CF patients, which is close to 50 years today, is expected to continue to increase in the future with the rapid expansion of newborn screening (NBS) for CF worldwide over the past decade and with the recent advent of cystic fibrosis transmembrane conductance regulator (CFTR) modulator therapies [3]. The frequency and severity of pulmonary complications increase with age [4]. The serratus anterior plane block (SAPB) was described in 2013 by Blanco et al. who presented it as an alternative to other regional anesthetic techniques in patients undergoing breast surgery [5]. While the SAPB has been described in adults as an adjunct to general anesthesia or as primary anesthetic technique for breast surgery, it has not been widely utilized as primary anesthetic technique in the pediatric population or in the management of patients with CF undergoing thoracic surgery [5]. We describe the utilization of this regional technique for a pediatric patient who presented for urgent thoracic intervention.

## Case presentation

**Figure 1:**
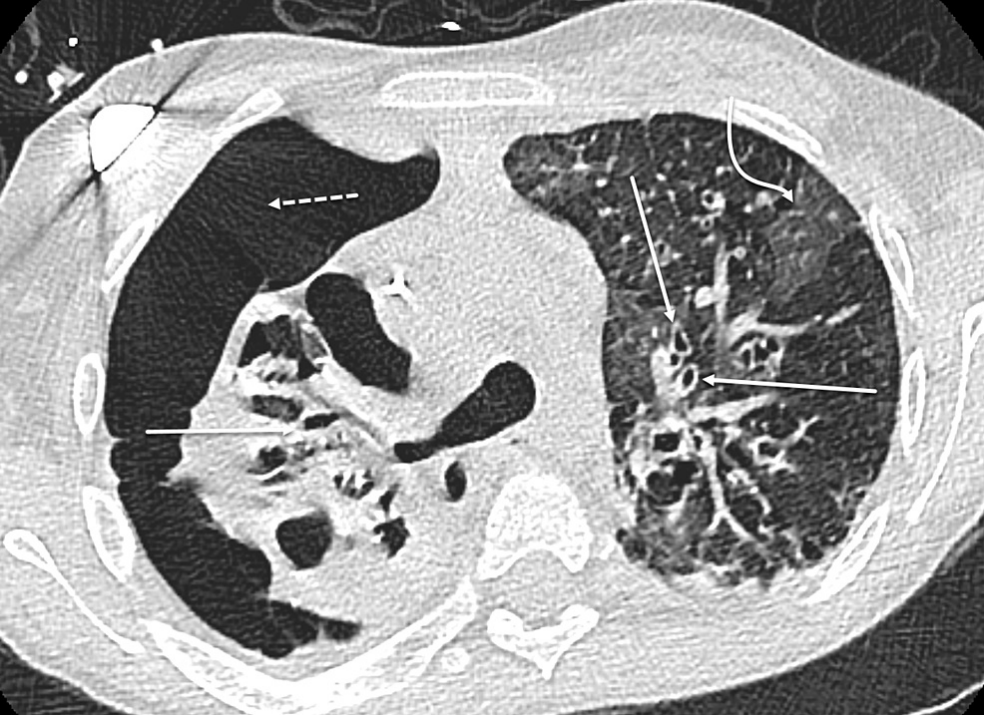
CT image chest without contrast. Multiseptated large right pneumothorax (dotted arrow), septations not shown. Advanced findings of CF with bilateral bronchiectasis (solid arrows) and concomitant ground glass infection (curved arrow). CF, cystic fibrosis

We present an 11-year-old, 30 kg, Hispanic female with severe CF who presented with new-onset chest pain and acute-on-chronic respiratory failure. At home, she was on daily inhalers and was maintained on bilevel positive airway pressure (BiPAP) at 18/9 cm H2O at night. Chest radiography and CT scan revealed a large right-sided pneumothorax with mild tension physiology (Figure 1). Respiratory cultures grew Pseudomonas aeruginosa, Achromobacter xylosoxidans, and Staphylococcus aureus. Despite treatment with a chest tube and high flow nasal cannula (HFNC) of 22 L/min at 70% fraction of inspired oxygen (FiO2), the pneumothorax persisted. She also remained tachypneic at a rate of 42 breaths/min. Due to concern for worsening respiratory function, she was scheduled to undergo video-assisted thoracic surgery (VATS). The patient and her family expressed concern over the possibility of prolonged ventilator dependence after surgery as this had occurred previously.

**Figure 2:**
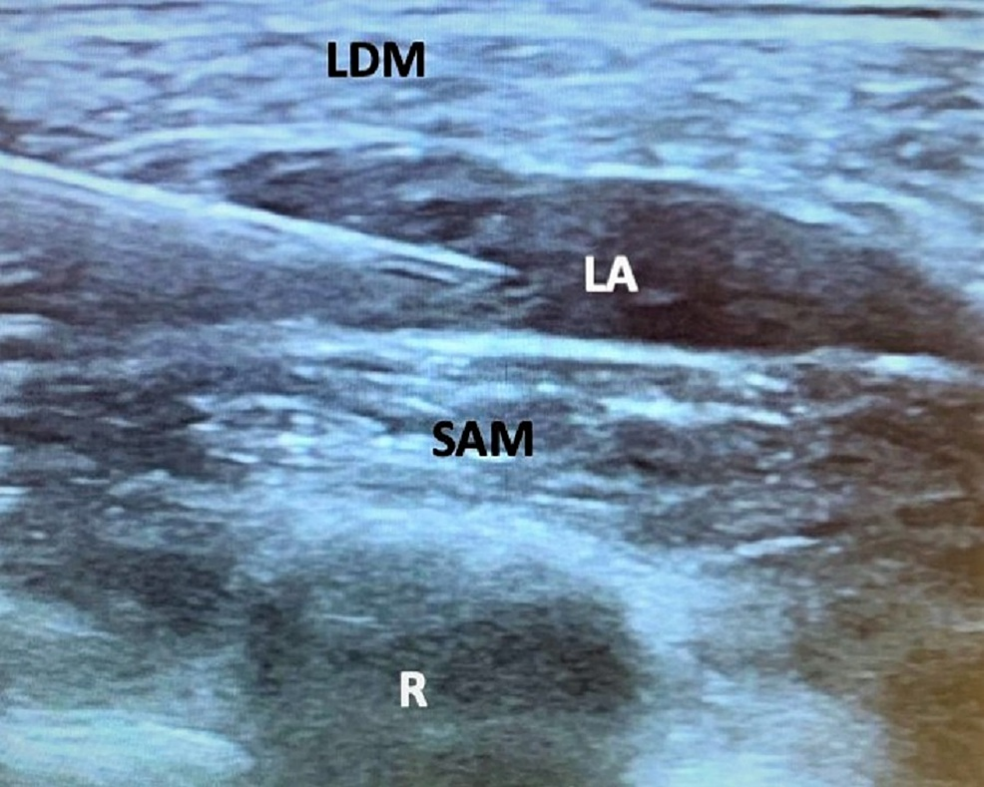
Ultrasound image of SAP block. SAP, serratus anterior plane; LDM, latissimus dorsi muscle; SAM, serratus anterior muscle; LA, local anesthetic; R, rib

After discussion with the surgeon and the family, we planned for SAPB with sedation. The patient was pre-medicated with midazolam (2 mg) and glycopyrrolate (0.4 mg). Sedation was provided with incremental doses of IV ketamine totaling 65 mg (~2 mg/kg) and dexmedetomidine infusion at 0.5-1 mcg/kg/h. For the SAPB, a 22-gauge, 50 mm echogenic needle was used under ultrasound guidance to locate the serratus anterior plane (SAP) at a depth of 1 cm from the skin. There, 20 mL of 0.2% ropivacaine (1.3 mg/kg) and 40 mcg of dexmedetomidine (1.3 mcg/kg) were injected (Figure 2). Airway management consisted of 22 L/min HFNC at 70% FiO2, with intermittent continuous positive pressure applied via face mask. The patient tolerated the procedure well and was assessed to be under deep sedation for the majority of the case. Over the next 24 h in the ICU, she continued HFNC and required four doses of IV morphine (0.1 mg/kg per dose), with an average pain score of four out of 10. After 2.5 weeks, the chest tube was removed and she was successfully weaned to 2 L/min nasal cannula (NC) prior to being transferred to the inpatient ward.

## Discussion

Patients with CF typically present with recurrent pulmonary infection, productive cough, hypoxemia, viscous secretions, hyperinflation of the lung, obstructive lung disease, and pneumothorax. The median age for the development of pneumothorax in CF patients is 20 years [6]. The development of pneumothorax is associated with increased morbidity and mortality in these patients, particularly when the first pneumothorax appears at a younger age [6-7]. Our patient’s presentation with pneumothorax at 11 years of age was indicative of her advanced disease. Her risk factors for pneumothorax included a history of acute on chronic pulmonary infection with P. aeruginosa, home BiPAP use, and administration of tobramycin and dornase alfa [6-7].

The complexity of the respiratory disease found in CF patients poses a challenge for anesthesiologists, intensivists, and surgeons as well as the pulmonologists who provide long-term care. The progressive nature of the lung disease in patients with CF places them at increased likelihood of requiring prolonged ventilatory support during the postoperative period. Thoracic epidural anesthesia, intercostal nerve blocks, and paravertebral blocks are other regional techniques that are used for VATS [8-9]. In this case, the utilization of the SAPB provided a surgical level of anesthesia for VATS and facilitated the use of deep sedation with natural airway rather than general anesthesia with an endotracheal tube. The SAPB also provided enough residual analgesia to allow for a reduced narcotic requirement in the first 24 h post-surgery.

## Conclusions

Patients with CF present with many respiratory complications. The severity of their disease may place them at risk of prolonged ventilatory support after general anesthesia. In our case, the use of SAPB mitigated this risk by providing a surgical level of anesthesia to the anterolateral thoracic wall and allowing for spontaneous ventilation during this invasive procedure that would otherwise require general anesthesia with an endotracheal tube. We demonstrate that with careful patient selection and an experienced pediatric surgeon, the SAPB is a valuable tool in the intraoperative as well as postoperative management of children with severe CF who present for VATS. 

## References

[1] Fitzgerald M, Ryan D (2011). Cystic fibrosis and anaesthesia. Contin Educ Anaesth Crit Care Pain.

[2] Stern RC (1997). The diagnosis of cystic fibrosis. N Engl J Med.

[3] Scotet V, L'Hostis C, Férec C (2020). The changing epidemiology of cystic fibrosis: incidence, survival and impact of the <i>CFTR</i> gene discovery. Genes (Basel).

[4] (2021). 2019 Patient Registry Annual Data Report- Cystic Fibrosis Foundation. https://www.cff.org/Research/Researcher-Resources/Patient-Registry/2019-Patient-Registry-Annual-Data-Report.pdf.

[5] Blanco R, Parras T, McDonnell JG, Prats-Galino A (2013). Serratus plane block: a novel ultrasound-guided thoracic wall nerve block. Anaesthesia.

[6] Flume PA, Strange C, Ye X (2005). Pneumothorax in cystic fibrosis. Chest.

[7] Kioumis IP, Zarogoulidis K, Huang H (2014). Pneumothorax in cystic fibrosis. J Thorac Dis.

[8] Sugiyama T, Kataoka Y, Shindo K, Hino M, Itoi K, Sato Y, Tanaka S (2021). Retrolaminar block versus paravertebral block for pain relief after less-invasive lung surgery: a randomized, non-inferiority controlled trial. Cureus.

[9] Kumar K, Basker S, Jeslin L, Karthikeyan C, Matthias A (2011). Anaesthesia for pediatric video assisted thoracoscopic surgery. J Anaesthesiol Clin Pharmacol.

